# Perceived Social Support from Different Sources and Adolescent Life Satisfaction Across 42 Countries/Regions: The Moderating Role of National-Level Generalized Trust

**DOI:** 10.1007/s10964-021-01441-z

**Published:** 2021-05-15

**Authors:** Shanshan Bi, Gonneke W.J.M. Stevens, Marlies Maes, Maartje Boer, Katrijn Delaruelle, Charli Eriksson, Fiona M. Brooks, Riki Tesler, Winneke A. van der Schuur, Catrin Finkenauer

**Affiliations:** 1 Department of Interdisciplinary Social Science, Utrecht University, Padualaan 14, 3584 CH Utrecht, The Netherlands; 2 Department of School Psychology and Development in Context, KU Leuven, Tiensestraat 102, 3000 Leuven, Belgium; 3 Research Foundation – Flanders (FWO), Egmontstraat 5, 1000 Brussels, Belgium; 4 Department of Public Health and Primary Care, Ghent University, Corneel Heymanslaan 10, 9000 Ghent, Belgium; 5 Department of Sociology, Ghent University, Korte Meer 5, 9000 Ghent, Belgium; 6 Department of Public Health Sciences, Stockholm University, Sveavägen 160, SE 106 91 Stockholm, Sweden; 7 Faculty of Health, University of Technology Sydney, Ultimo, NSW Australia; 8 Department of Health Systems Management, Ariel University, Ariel, Ramat HaGolan St 65, Israel

**Keywords:** Different sources of perceived social support, Life satisfaction, National-level generalized trust, Early adolescents, Multilevel regression analysis

## Abstract

Although previous research established a positive association between perceived social support and adolescent life satisfaction, little is known about the relative importance of different sources of support for adolescent life satisfaction and cross-country variations in this respect. Using large-scale representative samples from the 2017/18 Health Behaviour in School-aged Children (HBSC) study, this study examined to what extent the association between social support and life satisfaction in early adolescence varied across different social sources and countries. Also, it examined whether cross-country variations are explained by national-level generalized trust, a sociocultural factor that shapes adolescent socialization. National-level data were linked to data from 183,918 early adolescents (*M*_*age*_ = 13.56, *SD* = 1.63, 52% girls) from 42 European and North American countries/regions obtained from HBSC. Multilevel regression analyses yielded a positive association between support from different sources and life satisfaction. The strongest associations were found for support from families, followed by teachers and classmates, and weakest for support from friends. Associations varied across different countries/regions. National-level trust amplified the association between perceived classmate support and adolescent life satisfaction. The revealed cross-country differences open avenues for future cross-cultural research on explanations for cross-cultural differences in the association between social support from different sources and life satisfaction in early adolescence.

## Introduction

Perceived social support from different sources is beneficial for adolescent well-being (Chu et al., 2010). It protects adolescents from internalizing symptoms (e.g., depression, anxiety, and loneliness; Cavanaugh & Buehler, 2016; Rueger et al., 2016) and promotes positive feelings (e.g., hope, well-being, and security; Archer et al., 2019; Chu et al., 2010). Also, a large body of research found a positive association between perceived social support and adolescents’ life satisfaction (e.g., Jiménez-Iglesias et al., 2017). Whether the association varies across sources of social support and across different countries has received less empirical attention. Moreover, it remains unclear whether the sociocultural context in which adolescents are socialized, such as national-level generalized trust, plays a moderating role in the association between perceived social support and adolescents’ life satisfaction. Because adolescence is characterized by rapid changes in social networks and sociocultural contexts may affect the perception of social support, identifying sources of support that are most likely to boost adolescents’ life satisfaction in different countries is crucial. Answering these questions will enhance the scientific understanding of the association between perceived social support and adolescents’ life satisfaction and pave the way toward future interventions. The present study used large-scale representative samples from 42 countries/regions to investigate the associations between different sources of social support and early adolescents’ life satisfaction, whether these associations differ across countries, and the moderating role of national-level generalized trust.

### Perceived Social Support and Adolescent Life Satisfaction

Perceived social support refers to the extent to which adolescents believe that they can receive support from sources in their social environment (i.e., families, friends, teachers, and classmates), particularly when they need help (Bokhorst et al., 2010; Jiménez-Iglesias et al., 2017). Social support in adolescence is fundamental to many developmental processes, such as coping with stress and adversity (Finkenauer et al., 2019) and thriving and personal growth throughout the life-course (Deci & Ryan, 2000). Research shows that children, adolescents, and adults who perceive that they are cared for and valued by people in their social environments, and who experience more supportive and rewarding relationships have better mental and physical health (Demaray et al., 2005) and higher levels of subjective well-being (Chu et al., 2010). Life satisfaction—the degree to which a person positively evaluates the overall quality of their life as-a-whole (Veenhoven, 1996)—is considered a hallmark for mental and physical health and resilience in adolescence (Gilman & Huebner, 2003). Adolescents with higher life satisfaction receive more social support (Proctor et al., 2009). On a daily basis, adolescents simultaneously receive support from different sources in their social environments, yet not all sources of social support may be equally important for adolescent life satisfaction (Suldo & Huebner, 2006). Consistent with this suggestion and in accordance with the ecological model (Bronfenbrenner, 1979), research found that the association between perceived social support and adolescent well-being may differ across the source of support (Rueger et al., 2016). Because adolescence is a period marked by rapid changes and growth, different sources may provide different types of support during adolescence and adolescents may desire support from different sources (Malecki & Demaray, 2003). Thus, consideration of different sources of support, including both adults and peers as well as the family and school context, is important to better understand the association between social support and life satisfaction in adolescence.

Given the importance of parents, perceived support from parents and family may show a stronger relation with adolescent life satisfaction than perceived support from other sources. Children and adolescents rely on their parents for emotional and instrumental support (e.g., love, financial support), and when parental support is available, adolescents experience less stress, which bolsters mental health (Auerbach et al., 2011; Rueger et al., 2016). Consistent with this suggestion, studies found that perceived support by parents is more important to adolescents’ life satisfaction than perceived support by friends, teachers, and/or classmates (e.g., Jiménez-Iglesias et al., 2017).

Nevertheless, as school becomes more important and adolescents desire more autonomy from parents (Steinberg & Silk, 2002), perceived support by other sources may become increasingly important (i.e., friends, teachers, and classmates). Consistent with this suggestion, some studies found that perceived support from friends was more conducive to adolescents’ well-being than perceived support from parents, teachers, and/or classmates (Bokhorst et al., 2010; Leme et al., 2015). A meta-analysis of American studies showed that support from teachers and school personnel showed a stronger association with children’s and adolescents’ well-being than support from families and other sources (Chu et al., 2010). Thus, although there is robust evidence that perceived social support from different sources is positively associated with life satisfaction in adolescence, the relative importance of support from different sources for life satisfaction in adolescence remains unclear.

### Cross-Country Variations in the Association Between Perceived Social Support and Life Satisfaction: The Moderating Role of National-Level Generalized Trust

Although models on social support often assume global validity and applicability, sociocultural factors, norms, and values may moderate the relative importance of support from different sources for adolescents’ life satisfaction (Cohen et al., 2000; Oishi et al., 1999). To maximize benefits and minimize potential harm to individuals, groups, and communities, research needs to document and explore differences and similarities across countries to explore cross-country variation (van de Vijver, 2013). Indeed, research suggests that the association between different sources of perceived social support and adolescent life satisfaction may vary across countries. For instance, cross-sectional studies have found that in some countries, such as the United States, Portugal, and Spain, adolescents reported that perceived family support (or parental support) was more strongly associated with life satisfaction than perceived support from other social sources (Jiménez-Iglesias et al., 2017; Siddall et al., 2013; Stewart & Suldo, 2011). In other countries, such as the Netherlands and Brazil, perceived social support from friends was equally or even more strongly linked to life satisfaction than perceived social support from other sources (Bokhorst et al., 2010; Leme et al., 2015). In Norway, teacher support and classmate support were found to be almost equally important for life satisfaction as parental support (Danielsen et al., 2009). Given these results from different studies, it seems reasonable to assume that there are cross-country differences in the association between different sources of perceived social support and adolescent life satisfaction. However, the few existing studies that have examined this question only use data from a small number of countries simultaneously. To examine the generalizability of these findings and to provide leverage points for future interventions targeting adolescent life satisfaction, large-scale representative samples are necessary to investigate the extent to which sources of social support are important for early adolescents’ life satisfaction and whether these associations vary across countries.

National-level generalized trust is a sociocultural factor that has been suggested to affect people’s lives and health through its socialization processes (Balliet & Van Lange, 2013; Schneier, 2012). National-level generalized trust refers to the extent to which a country fosters a safe and reliable social context for its citizens. Specifically, it represents a shared value that portrays the overall interpersonal trust climate of social members in a specific society (i.e., “the overall perception that people can be trusted”; Balliet & Van Lange, 2013). Prior research showed that national-level trust characterizes societies where public institutions act competently and are accessible to all citizens, and where people cooperate and express solidarity with one another (Zak, 2007). Compared to lower-trust countries, higher-trust countries, such as Norway, Sweden, and the Netherlands, are characterized by higher degrees of social cohesion. They are also more likely to invest in human security and social safety-nets, which may reduce anxiety and fear about the behavior of others, and thereby contribute to people’s life satisfaction (Rostila, 2007).

The current study does not focus on the overall impact of national-level trust on adolescent life satisfaction. Rather, it focusses on its moderating role in the association between perceived social support and adolescent life satisfaction. According to the ecological theory (Bronfenbrenner, 1979), factors in adolescents’ microsystem (e.g., social support from different sources) and macrosystem (e.g., national-level trust) interact to shape adolescents’ developmental outcomes. Consequently, it can be expected that adolescents’ life satisfaction varies as a function of factors in both social contexts. So far, the question whether national-level trust might amplify or mitigate the association between perceived social support and adolescent life satisfaction has not been investigated empirically.

Derived from the sociocultural theory (Vygotsky, 1978), it can be assumed that each source of support is more impactful for people living within higher-trust social climates than those within lower-trust social climates. Specifically, when children and adolescents observe and witness that people in their environment are trusting and cooperating, they internalize the beliefs and attitudes towards others as prosocial and supportive, and extend those beliefs and attitudes to their own interactions with others (Van Lange, 2015). Thus, especially in higher-trust countries, all sorts of reciprocal interactions with others may become a strong signal for a secure feeling that “I can trust and rely on other people”, and as such may be more strongly linked to life satisfaction than in lower-trust countries (Knack, 2001). Conversely, in lower-trust countries, people witness and experience more negative interactions between others, such as bullying, burglary, and robbery, which, when internalized, reinforce a negative feeling that “I cannot trust other people.” This may give rise to more anxiety and insecurity in the interactions with other people, thereby potentially undermining the positive effects of social support on people’s life satisfaction (Zak, 2007). Thus, in the sociocultural perspective, the link between perceived social support and life satisfaction should be stronger in higher-trust countries than in lower-trust countries.

In contrast, the resilience model (Fergus & Zimmerman, 2005) applied to the current study predicts that different sources of support should be especially impactful in lower-trust countries. Specifically, in lower-trust countries, each source of social support may compensate for the health-damaging contextual effects of lack of social cohesion, lack of effective institutions, economic inequality, and as such may help adolescents to cope with adversity and stress. In this regard, in lower-trust societies, social support from parents, friends, teachers, and/or classmates could make an important difference in adolescents’ life satisfaction (Fergus & Zimmerman, 2005; Masten, 2001). In contrast, in higher-trust countries, adolescents have access to a wide range of social support sources, so specific sources may not necessarily add to adolescents’ perceived level of social support, and thus their life satisfaction. Thus, from the resilience perspective, the link between perceived social support and adolescent life satisfaction may be stronger in lower- than in higher-trust countries.

## Current Study

The current study investigates associations between perceived social support from different sources in early adolescents’ social environment (i.e., families, friends, teachers, and classmates) and their life satisfaction, as well as cross-country variations and the moderating role of national-level trust in these associations. Data are obtained from the 2017/18 Health Behaviour in School aged Children (HBSC) study across 42 countries/regions with 183,918 adolescents to test four hypotheses. Based on the current literature, first, perceived social support is hypothesized to be positively associated with life satisfaction in early adolescence. Second, the strength of this association is expected to differ across different sources of support. Third, cross-country variations in the association between each source of perceived social support and adolescent life satisfaction are expected. Fourth, it is hypothesized that national-level trust moderates this association, but the direction of the moderation remains unclear. To facilitate transparency, this study was pre-registered on the AsPredicted platform (https://aspredicted.org/my3b4.pdf).

## Methods

### Study Population and Procedures

Individual-level data from the 2017/18 cycle of the HBSC study were used. This 2017/18 survey was conducted across 46 countries/regions in Europe and North America. Of these countries, 42 countries were included in the analysis (*N* = 227,681). Latvia and Azerbaijan were excluded as no data were available on perceived social support from teachers and classmates, or on family structure. Greenland was excluded due to missing national-level data (i.e., national-level trust). Also, Denmark was removed from the sample, because two friend support items had a correlation of 1, which caused convergence problems in one of the analyses. Appropriate ethical approval for the survey was obtained at the national-level. All countries (or regions) must comply with the international standard protocol to guarantee the cross-country comparability of the data (Inchley et al., 2020) and used cluster sampling to randomly select schools and classes in order to generate a random sample of 11-, 13-, and 15-year-olds. Schools, parents (or guardians), and children were provided with age-appropriate information to ensure they all fully understood the main goal, the content, and the anonymous procedure of the HBSC survey.

This study included only individuals with complete data on all analysis measures (*N* = 183,918). Of the participants, 52% were girls and the mean age was 13.56 years old (*SD* = 1.63). Missingness was spread across variables: age (0.7%); family structure (3.7%); family affluence (5.2%); perceived family support (8.0%), perceived friend support (5.2%), perceived teacher support (6.1%), perceived classmate support (5.6%), and life satisfaction (1.7%). For the individual-level continuous variables, an independent t-test was used to compare the means of each variable between the group with missing and the group with complete data. Cohen’s *d* was computed to calculate the effect size of the mean differences for each variable. For individual-level categorical variables, chi-square (χ^2^) tests were used, and subsequently Cramer’s *V* for effect sizes were computed. Compared to excluded adolescents, included adolescents were significantly more likely to be older (*M*_*age*_ = 13.56 vs. *M*_*age*_ = 13.25), female (52% vs. 45%), living with both biological parents (74% vs. 66%), and scored higher on family affluence (0.50 vs. 0.49), perceived family support (5.71 vs. 5.49), perceived friend support (5.31 vs. 5.04), perceived teacher support (3.80 vs. 3.79), perceived classmate support (3.84 vs. 3.81), and life satisfaction (7.77 vs. 7.68). Following Cohen’s conventional criteria for effect sizes (Cohen, 1992), all the effect sizes of the mean difference of each variable between two groups were small (0.01 < Cohen’s *d* < 0.19; 0.05 < Cramer’s *V* < 0.07), as t-test with a Cohen’s *d* below 0.2 or χ^2^ test with Cramer’s *V* below 0.1 indicates a small effect size of mean difference.

### Measures

Individual-level data on perceived social support, adolescent life satisfaction, and individual demographic characteristics were obtained from the 2017/18 HBSC study. National-level data on national-level trust and national characteristics were obtained from internationally authoritative online sources. In addition, individual demographic characteristics and national characteristics were added in the regression analyses, as these characteristics may be associated with perceived social support (De Looze et al., 2018), adolescent life satisfaction (Zaborskis et al., 2019), and/or national-level trust (Yamagishi, 2011).

#### Individual-Level Variables

##### Perceived Social Support

*Family support* and *friend support* were measured using the 8-item family and friend subscales of the Multidimensional Scale of Perceived Social Support (MSPSS; Zimet et al., 1988). On a 7-point scale ranging from 1 (*strongly disagree*) to 7 (*strongly agree*), adolescents rated how much they felt supported by their families/friends (i.e., “My family/friend(s) really tries to help me”, “I get the emotional help and support I need from my family”, “I can talk about my problems with my family/friends”, “My family is willing to help me make decisions”, “I can count on my friends when things go wrong”, and “I have friends with whom I can share my joys and sorrows”). Higher scores indicated higher levels of perceived support from families/friends. The scale is reliable and valid as has been shown in studies among various adolescent samples from different countries (e.g., Başol, 2008; Canty-Mitchell & Zimet, 2000; Edwards, 2004). In this study, Cronbach’s alphas were 0.94 for family support and 0.92 for friend support.

*Teacher support* and *classmate support* were assessed using the 3-item Teacher Support Scale and the 3-item Classmate Support Scale (Torsheim et al., 2000). On a 5-point scale ranging from 1 (*strongly agree*) to 5 (*strongly disagree*), adolescents indicated the extent to which they experienced their teachers/classmates as supportive (i.e., “I feel that my teachers accept me as I am”, “I feel a lot of trust in my teachers”, “I feel that my teachers care about me as a person”, “The students in my class(es) enjoy being together”, “Most of the students in my class(es) are kind and helpful”, and “Other students accept me as I am”). For the purpose of this study, the items were recoded, such that higher scores indicated higher level of perceived support from teachers and classmates. The cross-country reliability and validity of the teacher and classmate measure has been demonstrated previously (Torsheim et al., 2012). In this study, Cronbach’s alphas were 0.83 for teacher support and 0.77 for classmate support.

##### Adolescent Life Satisfaction

*Adolescent life satisfaction* was measured using the Cantril (1965) ladder. Adolescents rated their satisfaction with life on a scale from 0 (*worst possible life*) to 10 (*best possible life*) to indicate how satisfied they feel with their lives. Higher scores indicated higher level of life satisfaction. Previous research has shown sufficient test-retest reliability among adolescents (Levin & Currie, 2014). Also, the Cantril Ladder has been well validated for use with adolescent populations, and associations with school success and well-being indicators were found (Jovanović, 2016).

##### Demographic Characteristics

*Age* was calculated by the year of survey administration minus each participant’s year of birth. Gender was reported by a survey item (i.e., “Are you a boy or a girl?”) and was coded as *female* (1) or *male* (0). *Family structure* was measured by one question, asking adolescents to report “Who resides in the home where you live all or most of the time, including father, mother, stepfather, stepmother, and others?”. Because adolescents living with both biological parents showed higher life satisfaction than adolescents living in other family structures (Bjarnason et al., 2012), the response answers were coded as *living with both biological parents* (1) or *living with other family structures* (0). *Family affluence* was measured using the Family Affluence Scale (FAS; Torsheim et al., 2016), which is an indicator of young people’s socio-economic status, and comprises of six items on material assets in the family. Scale scores were calculated by summing up the scores of all six items. These sum-scores were transformed into proportional ranks that denote respondents’ relative family affluence given their residential country (Elgar et al., 2013). The FAS scores were in a 0–1 range with a mean of 0.5 in each country.

#### National-Level Variables

##### National-Level Generalized Trust

Data on *national-level generalized trust* were obtained from the data used to harmonize all available survey responses in the Harmonized Trust Database (HTD) compiled by the Global Trust Research Consortium (GTRC; https://globaltrustresearch.wordpress.com/results/). In the HTD, most surveys asked respondents to rate items such as “Most people can be trusted” or “You cannot be too careful” on different Likert scales, including 1–4 point scales, 1–5 point scales, 1–10 point scales and 0–10 point scales. GTRC rescaled all the available survey responses to a 0–100 score (the percentage of respondents choosing “Most people can be trusted”). The historical average scores for 155 countries were calculated by applying mega-analysis, which was an effective technique to deal with the differences between datasets in the mode of data collection used and differences in sampling frames and response rates (Bekkers, 2018). Compared to the national-level trust scores from single datasets, the historical average scores released by GTRC are better able to represent the nationally shared value, namely generalized trust at the national level, because the scores not only included multiple datasets, but also obtained the highest possible number of respondents in every available country. The national-generalized trust scores for all 42 countries/regions included in the HBSC study were selected for the current study. Higher trust scores indicate a higher degree of generalized trust on national level.

##### National Characteristics

Data on *national wealth*- Gross Domestic Product (GDP) per capita in purchasing power parity (PPP)-were obtained from the World Bank of International Comparison Program database for 2017. GDP in PPP is a standardized measure that takes into account countries’ differences in affluence. For the purpose of interpretation, the raw data on national wealth were divided by 1 trillion. Data on *income inequality* were obtained from the World Bank of GINI index which ranged from 0 (everyone has equal income) to 100 (one person has all the income). Of these 42 countries, some countries did not update their GINI coefficients in 2017. The GINI index of these countries used the data which were closest to 2017, as the change of the GINI coefficient for each country in the last decade was marginal in general. Data on *government expense* (% GDP) were also obtained from the World Bank for 2017.

In addition, regarding data from the United Kingdom (UK), the individual-level data were obtained from three regions (Scotland, Wales, and England), while the national-level data represented one general score for the UK on each variable (i.e., national-level trust and national characteristics). Similarly, in Belgium, a separate individual-level dataset was available for the French Belgium region and the Flemish Belgium region, while the national-level data represented one general score for Belgium on each variable. In this study, when the multilevel regression analyses were conducted, Scotland, Wales, and England were assigned the UK scores for the national-level data, and the French Belgium and Flemish Belgium were assigned the Belgium scores for the national-level data.

### Measurement Invariance

To examine the relative importance of different sources of perceived social support in adolescent life satisfaction across countries/regions, items of the measures used should be interpreted in the same way by participants from those different countries/regions. This is the case when the factor structure of the measurement model is approximately equal across countries/regions. Therefore, prior to the analyses, measurement invariance (MI) across countries/regions was examined using multigroup confirmatory factor analysis (CFA) with the “lavaan” and “semTools” packages. A baseline model was estimated that included all four factors (i.e., sources of support) and correlations between these factors. Subsequently, a three-step method was applied, testing configural (i.e., free intercepts and loadings across countries), metric (i.e., free intercepts and constrained loadings), and scalar invariance (i.e., constrained loadings and intercepts). Model fit was evaluated based on comparative fit index (CFI), Tucker–Lewis index (TLI) and the root mean square error of approximation (RMSEA). Models with CFI/TLI > 0.9 and with RMSEA < 0.08 provided acceptable model fit (van de Schoot et al., 2012). Moreover, invariance could be met, when the model fit of the metric and scalar model decreased CFI values (ΔCFI) with less than 0.010, and increased RMSEA values (*ΔRMSEA*) with less than 0.015 compared to the configural and metric model, respectively (Chen, 2007; Cheung & Rensvold, 2002). According to these fit indices, metric invariance, but not scalar invariance, has been established (ΔCFI = −0.007, ΔRMSEA = 0.003; see Appendix 1 for more information). These findings indicate that it is valid to proceed with the comparisons of regression coefficients across countries/regions, especially for the regression model with latent variables (Boer et al., 2018; Chen, 2007).

### Data Analysis

For the descriptive results, data were analyzed (Pallant, 2016). Means and standard deviations of all study variables were computed to investigate the sample characteristics. Bivariate correlations among individual characteristics (i.e., gender, age, family structure, and family affluence), all the perceived social support measures, and adolescent life satisfaction were computed.

Multilevel regression analyses were carried out using the “lme4” package for a multilevel regression model in R.4.0.3 (Finch et al., 2014; R Core Team., 2020). Two-level linear regression models investigated (1) overall associations between each source of perceived social support and adolescent life satisfaction; (2) differences in associations between perceived social support and adolescent life satisfaction across the four sources of social support; (3) cross-country variations in the associations between each source of perceived social support and adolescent life satisfaction; (4) the moderating role of national-level trust in these associations. Individual- and national-level variables were added to the models using a stepwise approach. Individuals were clustered within countries/regions (*n* = 42). Individual- and national-level variables were added to the models using a stepwise approach. To facilitate consistency of data scaling and interpretation of the moderation effects and to reduce multicollinearity, age, family affluence, and the values of perceived social support were group-mean centered, and national-level trust and national characteristics were grand-mean centered.

Model 0 included all social support measures and individual demographic characteristics simultaneously to test whether the association between perceived social support and life satisfaction varied across countries/regions while slopes were fixed between countries/regions to the average value. Model 0 served as a baseline for the subsequent models. Model 1 included random slopes for family (1a), friend (1b), teacher (1c), and classmate support (1d) to assess whether there were significant variations between countries in the links between each source of perceived social support and life satisfaction (while adjusting for individual demographic characteristics and the other three sources of support). To investigate whether there were national-level differences in the association between perceived social support from the specific source and adolescent life satisfaction, this study pre-registered to use the alpha level (*p* < 0.05) of the variance of the random slope. However, the alpha level of this variance cannot be obtained from the lme4-package that was used for the multilevel regression models in R version 4.0.3. Therefore, as an alternative, the ranova() function of the lmerTest package was used. With this function, the fit of the model with the random slope (Model 1a-d) was compared with that of the model without the random slope (Model 0, see Appendix 2) using a likelihood ratio-test. If the χ^2^ test was significant (*p* < 0.05), it indicated that the specific random slope improved model fit, which implies that the association between perceived social support from the specific source and adolescent life satisfaction varied across countries/regions (Smeets & van de Schoot, 2019). Next, to construct the final model, several steps were taken. When random slopes improved the model fit for each source of social support, a model including all four random slopes simultaneously was examined (Model 1e). Then, the four national-level variables (Model 1f) were added, followed by a model that also included the four cross-level interactions between national trust and the social support sources (Model 1g). To avoid overcomplication, the non-significant variables (i.e., national characteristics and the three interaction terms) were removed for the final model (Model 2). Model 2 with the main and the moderating effects of national-level trust was the best fitting model.

To gain a better understanding of the relative importance of different sources of perceived social support in adolescent life satisfaction in each country/region, multivariate regression models were conducted using IBM SPSS Statistics (Version 26) predictive analytics software (IBM Corp., 2019; Pallant, 2016). First, the dataset was split into 42 groups by countries/regions and then multivariate regression analyses for life satisfaction were conducted. The individual demographic characteristics and all perceived social support measures were simultaneously entered in the model. In accordance with the new statistics movement (Cumming et al., 2012), standardized coefficients (βs) and 95% confidence interval (CI) for unstandardized coefficients (*Bs*) were reported in Table 4. The βs of the four sources of perceived social support enabled a comparison of the relative importance of these four sources between and/or within countries/regions. To ensure the results for multivariate regression with observed variables were reliable, a sensitivity analysis was conducted. More specifically, the analysis with latent, rather than observed, variables was repeated, using multigroup structural equation modeling (SEM) by country/region using the “lavaan” package in R.4.0.3 (R Core Team., 2020), while controlling for individual demographic characteristics. Then, the correlations between the βs for the multigroup SEM (effects based on latent variables that take into account country-specific measurement error) and the βs for the multivariate regression models (effects based on observed variables) were computed. The analyses based on observed variables versus latent variables provided almost identical results. This is due to the fact that the correlations between the βs from the multigroup SEM (Appendix 3) and the βs from the multivariate regression models with observed variables (Table 3) were significant and very high (perceived family support: *r* = 0.996, *p* < 0.01; perceived friend support: *r* = 0.981, *p* < 0.01; perceived teacher support: *r* = 0.979, *p* < 0.01; and perceived classmate support: *r* = 0.983, *p* < 0.01). These findings suggest that the analyses were not biased by the fact that observed variables were used instead of latent variables.

## Results

### Descriptive Results

As shown in Table 1, adolescents (*N* = 183,918) generally were satisfied with their lives (*M* = 7.77, *SD* = 1.87), with average life satisfaction scores ranging from 7.30 (Canada) to 8.57 (Kazakhstan). Despite the fact that life satisfaction was non-normally distributed, with skewness of −1.050 (*SE* = 0.006) and kurtosis of 1.370 (*SE* = 0.011), the violation of the normality assumption in linear regression models is acceptable and unlikely to have a significant impact on the results, because large representative data were used in this study (Schmidt & Finan, 2018). Also, given the considerable sample sizes both at the individual and the country-level, the consistent sampling procedures across countries, and the representative samples, the impact of outliers on the results is likely to be limited (Kline, 2017).

**Table 1:** Descriptive characteristics of the individual- and national-level variables (N _individual_ = 183,918; N _country_ = 42)

Sample	Individual-Level Characteristics								National-Level Characteristics			
Country	*N*	Girls (%)	Age (*M*)	Life satisfaction (*M*)	Perceived family support^a^ (*M*)	Perceived friend support^a^ (*M*)	Perceived teacher support^b^ (*M*)	Perceived classmate support^b^ (*M*)	National-level trust	National wealth^c^	Income inequality	Government expense
Albania	1453	0.57	13.54	8.12	6.39	5.50	4.30	4.21	19.04	0.04	33.20	23.87
Armenia	3274	0.55	13.54	8.39	5.93	5.55	4.12	4.34	26.57	0.04	33.60	22.63
Austria	3502	0.52	13.35	7.70	5.89	5.70	3.75	4.02	42.72	0.48	29.70	44.14
Belgium (Flemish)	3711	0.52	13.37	7.82	5.88	5.61	3.98	4.05	41.16	0.58	27.40	39.88
Belgium (French)	3257	0.52	12.86	7.66	5.90	5.52	3.93	3.90	41.16	0.58	27.40	39.88
Bulgaria	3953	0.52	13.58	7.84	4.60	4.57	3.71	3.62	30.90	0.15	40.40	32.21
Canada	8643	0.53	13.78	7.30	5.03	4.89	3.87	3.61	36.87	1.78	33.80	17.60
Croatia	4046	0.51	13.86	8.12	6.11	5.56	3.68	3.86	28.52	0.11	30.40	38.73
Czech Republic	10046	0.50	13.45	7.79	5.07	4.67	3.57	3.56	44.04	0.41	24.90	32.09
England	2715	0.48	13.45	7.46	4.92	4.50	3.76	3.64	41.53	3.04	34.80	37.17
Estonia	4401	0.51	13.83	7.73	5.93	5.32	3.72	3.83	38.39	0.04	30.40	35.10
Finland	2825	0.52	13.91	7.78	5.72	5.47	3.85	3.90	59.45	0.26	27.40	37.95
France	7391	0.52	13.34	7.65	5.83	5.51	3.82	3.85	36.03	3.00	31.60	47.52
Georgia	3472	0.53	13.49	7.99	5.29	5.90	4.00	3.91	31.21	0.05	37.90	22.99
Germany	3625	0.54	13.56	7.67	5.84	5.61	3.74	4.06	37.39	4.38	31.90	28.22
Greece	3638	0.51	13.85	7.53	6.01	5.73	3.74	3.62	31.38	0.31	34.40	46.80
Hungary	3440	0.53	13.57	7.58	6.20	6.02	3.64	3.76	38.31	0.29	30.60	42.32
Iceland	6299	0.51	13.65	7.61	5.65	5.32	4.04	3.97	47.61	0.02	26.80	30.16
Ireland	3215	0.51	13.42	7.55	5.36	5.28	3.87	3.97	45.82	0.38	32.80	24.22
Israel	5317	0.54	13.65	7.89	5.92	5.32	3.97	3.93	42.45	0.34	39.00	36.61
Italy	3807	0.53	13.69	7.60	5.97	5.63	3.75	3.93	31.13	2.53	35.90	42.81
Kazakhstan	3754	0.52	13.29	8.57	6.04	5.29	4.13	3.98	30.91	0.45	27.50	19.03
Lithuania	3550	0.51	13.73	7.90	5.80	5.25	3.61	3.64	34.85	0.10	37.30	31.09
Luxembourg	3335	0.51	13.58	7.66	5.84	5.63	3.66	3.96	39.26	0.07	34.90	38.23
Macedonia	3877	0.52	13.61	8.43	6.52	5.79	4.08	4.20	12.39	0.03	34.20	28.00
Malta	2180	0.54	13.32	7.37	5.95	5.59	4.01	3.99	19.24	0.02	29.20	35.07
Moldova	4136	0.51	13.58	8.23	5.77	4.96	3.89	3.88	18.23	0.03	25.90	26.80
Netherlands	4468	0.52	13.55	7.75	6.12	5.81	3.86	4.06	55.98	0.95	28.50	37.80
Norway	2366	0.52	13.09	7.92	6.24	5.72	4.17	4.19	57.23	0.33	27.00	39.02
Poland	4831	0.52	13.62	7.47	5.51	4.48	3.46	3.54	32.69	1.15	29.70	34.33
Portugal	5381	0.53	13.34	7.76	6.12	5.53	3.83	3.99	33.25	0.34	33.80	41.42
Romania	3786	0.52	13.19	8.35	6.13	5.26	3.75	3.93	16.24	0.53	36.00	31.58
Russia	3660	0.53	13.85	7.41	5.55	4.62	3.50	3.58	32.27	3.82	37.20	30.71
Scotland	4263	0.53	13.56	7.64	5.23	5.05	3.90	3.65	41.53	3.04	34.80	37.17
Serbia	3376	0.53	14.07	8.24	6.33	5.40	3.52	3.88	19.31	0.12	36.20	36.89
Slovakia	2971	0.52	13.45	7.60	5.81	5.22	3.56	3.66	30.43	0.17	25.20	38.98
Slovenia	5114	0.50	13.61	7.98	5.16	5.18	3.75	4.04	30.11	0.08	24.20	38.95
Spain	4088	0.52	13.64	8.09	6.10	6.04	3.74	3.94	37.14	1.84	34.70	18.83
Sweden	3462	0.52	13.69	7.47	6.06	5.68	4.10	3.93	58.07	0.53	28.80	31.68
Switzerland	6647	0.50	13.44	7.67	5.97	5.69	3.87	3.99	49.03	0.57	32.70	17.33
Ukraine	5654	0.54	13.49	7.66	5.67	4.90	3.53	3.50	36.56	0.50	26.00	33.58
Wales	10989	0.51	13.70	7.62	5.32	5.04	3.70	3.60	41.53	3.04	34.80	37.17
Mean	4379	0.52	13.56	7.77	5.71	5.31	3.80	3.84	36.14	0.87	31.74	33.49

Life satisfaction was measured using a scale from 0 to 10

*N* sample size, *M* mean

^a^Perceived family support and perceived friend support were measured using a 7-point scale

^b^Perceived teacher support and classmate support were measured using a 5-point scale

^c^The raw scores of national wealth were divided by 1 trillion

Regarding social support, perceived family support means ranged from 4.60 (Bulgaria) to 6.52 (Macedonia), perceived friend support means ranged from 4.48 (Poland) to 6.04 (Spain), perceived teacher support means ranged from 3.46 (Poland) to 4.30 (Albania), and perceived classmate support means ranged from 3.50 (Ukraine) to 4.34 (Armenia). Appendix 4 provides the graphical overviews of the mean scores and 95% confidence intervals (CI) of life satisfaction and each perceived social support measure by country.

In terms of national characteristics, the highest trust countries were in Scandinavia, with Finland, Sweden, and Norway ranking as the top three with trust scores of 59.45, 58.07, and 57.23, respectively. The lowest trust country was Macedonia with a trust score of 12.39. National wealth ranged from 0.02 (Iceland) to 4.38 (Germany). Income inequality ranged from 24.20 (Slovenia) to 40.40 (Bulgaria). Government expense ranged from 17.33% (Switzerland) to 47.52% (France). The correlations of national-level trust with national characteristics (*n* = 42) were not significant: national-level trust was not related to national wealth (*r* = 0.13, *p* = 0.39), income inequality (*r* = −0.26, *p* = 0.10), or government expense (*r* = 0.14, *p* = 0.36).

In Table 2, correlations among the individual-level variables are presented. Focusing on the links between life satisfaction and perceived social support from each source, life satisfaction had positive correlations with perceived family support (*r* = 0.28*, p* < 0.01), perceived friend support (*r* = 0.17, *p* < 0.01), perceived teacher support (*r* = 0.29*, p* < 0.01), and perceived classmate support (*r* = 0.29*, p* < 0.01). Moreover, perceived support from families, from teachers, and from classmates were negatively related to age and gender (male was the reference group), indicating that younger adolescents and boys were more likely to perceive social support from these three sources, compared to older adolescents and girls. In addition, younger adolescents and girls perceived more social support from friends than older adolescents and boys, although the strength of the association between age and friend support was almost zero (*r* = −0.01, *p* < 0.01). Family structure was positively associated with adolescent life satisfaction (*r* = 0.14*, p* < 0.01), family support (*r* = 0.10*, p* < 0.01), friend support (*r* = 0.06*, p* < 0.01), teacher support (*r* = 0.07*, p* < 0.01), and classmate support (*r* = 0.09*, p* < 0.01), indicating that adolescents living with both biological parents were more satisfied with their lives and more likely to perceive social support from all the sources. Family affluence had negligible to small positive associations with life satisfaction (*r* = 0.13*, p* < 0.01), family support (*r* = 0.06*, p* < 0.01), friend support (*r* = 0.06*, p* < 0.01), and classmate support (*r* = −0.01*, p* < 0.01), and a small negative association with teacher support (*r* = *−*0.05*, p* < 0.01).

**Table 2:** Correlations between individual-level variables (*N* = 183,918)

Variables	*M*	*SD*	1	2	3	4	5	6	7	8
1. Gender^a^	0.52	0.50								
2. Age	13.56	1.63	0.01*							
3. Family structure^b^	0.74	0.44	−0.01^**^	−0.04^**^						
4. Relative family affluence	0.50	0.29	0.00	0.00	0.15^**^					
5. Perceived family support^c^	5.71	1.72	−0.02^**^	−0.11^**^	0.10^**^	0.06^**^				
6. Perceived friend support^c^	5.31	1.73	0.10^**^	−0.01^**^	0.06^**^	0.06^**^	0.38^**^			
7. Perceived teacher support^d^	3.80	0.92	−0.02^**^	−0.25^**^	0.07^**^	−0.01^**^	0.20^**^	0.15^**^		
8. Perceived classmate support^d^	3.84	0.84	−0.07^**^	−0.11^**^	0.09^**^	0.05^**^	0.19^**^	0.24^**^	0.41^**^	
9. Life satisfaction	7.77	1.87	−0.07^**^	−0.18^**^	0.14^**^	0.13^**^	0.28^**^	0.17^**^	0.28^**^	0.28^**^

Life satisfaction was measured using a scale from 0 to 10

*M* mean, *SD* standard deviation

^a^Male is the reference group

^b^Living with other family structures is the reference group

^c^Perceived family support and perceived friend support were measured using a 7-point scale

^d^Perceived teacher support and perceived classmate support were measured using a 5-point scale

*p < 0.05, **p < 0.01

### Results for Multilevel Regression Analysis

National differences in the associations between the four sources of perceived social support and adolescent life satisfaction are presented in Table 3. Models 1a-d showed that the associations between the four sources of support—family support, friend support, teacher support, and classmate support—, and life satisfaction differed across countries, given that the models with the random slopes showed better model fit than those without random slopes (467.32 < χ^2^(2) < 3262.00, all *p*s < 0.001). The 95% prediction interval (PI) expresses that 95% of the regression coefficients of the sources of social support in the countries/regions are predicted to lie between the lower and upper bounds of the interval (Hox, 2010). Those intervals varied from negative to positive for the association between perceived family support and life satisfaction (M1a, 95% PI = −0.02 to 0.55), and for the association between perceived friend support and life satisfaction (M1b, 95% PI = −0.05 to 0.18). The prediction interval bounds were positive for the association between perceived teacher support and life satisfaction (M1c, 95% PI = 0.28 to 0.33), and for the association between perceived classmate support and life satisfaction (M1d, 95% PI = 0.09 to 0.59).

**Table 3:** Results of multilevel linear regression analyses of life satisfaction without and with cross-level interaction (N _individual_ = 183,918; N _country_ = 42)

	Model 1a (Random Slope for Family Support)				Model 1b (Random Slope for Friend Support)				Model 1c (Random Slope for Teacher Support)				Model 1d (Random Slope for Classmate Support)			
	*B*	*SE*	β	*t*-value	*B*	*SE*	β	*t*-value	*B*	*SE*	β	*t*-value	*B*	*SE*	β	*t*-value
Fixed effects (individual-level)																
Gender^a^	−0.21**	0.01	−0.06	−26.50	−0.22**	0.01	−0.06	−28.10	−0.21	0.01	−0.06**	−26.91	−0.21**	0.01	−0.06	−26.85
Age	−0.12**	0.00	−0.10	−48.67	−0.12**	0.00	−0.11	−50.27	−0.12	0.00	−0.11**	−49.87	−0.12**	0.00	−0.11	−49.68
Family structure^b^	0.30**	0.01	0.07	33.07	0.31**	0.01	0.07	33.89	0.31	0.01	0.07**	33.88	0.31**	0.01	0.07	33.77
Family affluence	0.64**	0.01	0.10	46.97	0.66**	0.01	0.10	48.19	0.67	0.01	0.10**	48.45	0.66**	0.01	0.10	48.34
Family support	0.27**	0.02	0.24	11.84	0.21**	0.00	0.19	81.00	0.20	0.00	0.18**	79.26	0.20**	0.00	0.18	79.31
Friend support	0.05**	0.00	0.04	18.25	0.06**	0.01	0.06	6.58	0.04	0.00	0.04**	17.62	0.05**	0.00	0.04	17.83
Teacher support	0.28**	0.00	0.14	58.91	0.30**	0.00	0.15	62.52	0.31	0.02	0.15**	17.60	0.30**	0.00	0.15	63.02
Classmate support	0.33**	0.01	0.14	63.15	0.34**	0.01	0.15	63.43	0.34	0.01	0.15**	65.14	0.34**	0.02	0.15	16.74

	Variance	*SD*	χ^2^(2)		Variance	*SD*	χ^2^(2)		Variance	*SD*	χ^2^(2)		Variance	*SD*	χ^2^(2)	
Random parameters																
Between-country variance	0.09	0.30			0.09	0.30			0.09	0.30			0.09	0.30		
Family support	0.02	0.15	3262.00^**^													
Friend support					0.00	0.06	562.46^**^									
Teacher support									0.01	0.11	467.32^**^					
Classmate support													0.02	0.13	585.73^**^	
Within-country variance	2.70	1.64			2.74	1.66			2.74	1.66			2.74	1.66		
95% prediction interval		[−0.02, 0.55]				[−0.05, 0.18]				[0.28, 0.33]				[0.09, 0.59]		

	Estimate				Estimate				Estimate				Estimate			
Model statistics																
AIC	705001.60				707701.13				707796.27				707677.85			
BIC	705133.19				707832.72				707927.85				707809.44			
Pseudo-*R*² (total)	0.25				0.22				0.21				0.21			
ICC	0.03				0.03				0.03				0.03			

	Model 1e (Random Slopes for All Social Support Variables)				Model 1f (Level-2 Main Effects)				Model 1g (With Interaction)				Model 2 (Best Fit)			
	*B*	*SE*	β	*t*-value	*B*	*SE*	β	*t*-value	*B*	*SE*	β	*t*-value	*B*	*SE*	β	*t*-value
Fixed effects (individual-level)																
Gender^a^	−0.21**	0.01	−0.06	−26.70	−0.21**	0.01	−0.06	−26.71	−0.21**	0.01	−0.06	−26.71	−0.21**	0.01	−0.06	−26.73
Age	−0.12**	0.00	−0.10	−48.50	−0.12*	0.00	−0.10	−48.50	−0.12**	0.00	−0.10	−48.52	−0.12**	0.00	−0.10	−48.52
Family structure^b^	0.29**	0.01	0.07	32.65	0.29**	0.01	0.07	32.64	0.29**	0.01	0.07	32.64	0.29**	0.01	0.07	32.65
Family affluence	0.63**	0.01	0.10	46.10	0.63**	0.01	0.10	46.10	0.63**	0.01	0.10	46.10	0.63**	0.01	0.10	46.10
Family support	0.27**	0.02	0.24	11.83	0.27**	0.02	0.25	11.83	0.27**	0.02	0.25	12.16	0.27**	0.02	0.25	11.84
Friend support	0.06**	0.01	0.05	8.47	0.06**	0.01	0.05	8.42	0.06**	0.01	0.05	8.47	0.06**	0.01	0.05	8.45
Teacher support	0.28**	0.01	0.14	19.86	0.28**	0.01	0.14	19.81	0.28**	0.01	0.14	19.80	0.28**	0.01	0.14	19.82
Classmate support	0.31**	0.02	0.14	17.63	0.31**	0.02	0.14	17.60	0.31**	0.02	0.14	20.15	0.31**	0.02	0.14	20.11
National-level																
Trust^c^					−0.00	0.00	−0.15	−1.44	−0.01**	0.00	−0.43	−3.25	−0.01*	0.00	−0.31	−2.88
National wealth					−0.03	0.03	−0.11	−1.11	−0.03	0.03	−0.12	−1.13				
Income inequality					−0.00	0.01	−0.02	−0.15	−0.00	0.01	−0.01	−0.14				
Government expense					−0.00	0.00	−0.12	−1.20	−0.00	0.00	−0.12	−1.21				
Cross-level interactions																
Family support × Trust^b^									0.00	0.00		1.52				
Friend support × Trust^b^									−0.00	0.00		−0.60				
Teacher support × Trust^b^									0.00	0.00		0.03				
Classmate support × Trust^b^									0.01**	0.00		3.53	0.00**	0.00		4.12

	Variance	*SD*			Variance	*SD*			Variance	*SD*			Variance	*SD*		
Random parameters																
Between-country variance	0.09	0.30			0.07	0.26			0.06	0.24			0.07	0.27		
Family support	0.02	0.15			0.02	0.15			0.02	0.14			0.02	0.15		
Friend support	0.00	0.04			0.00	0.04			0.00	0.04			0.00	0.04		
Teacher support	0.01	0.08			0.01	0.08			0.01	0.08			0.01	0.08		
Classmate support	0.01	0.11			0.01	0.11			0.01	0.09			0.01	0.09		
Within-country variance	2.68	1.64			2.68	1.64			2.68	1.64			2.68	1.64		

	Estimate				Estimate				Estimate				Estimate			
Model statistics																
AIC	703975.26				703979.48				703970.54				703964.71			
BIC	704228.31				704273.03				704304.57				704238.01			
Pseudo-*R*² (total)	0.25				0.25				0.25				0.25			
ICC	0.03				0.02				0.02				0.03			

Age, family affluence, and the values of perceived social support were group-mean centered. National-level trust and national characteristics were grand-mean centered. This chi-square measure refers to the likelihood ratio comparing the fit of the model (Models 1a-d) with the respective model without random slope. 95% prediction intervals were calculated by using *B* ± 1.96*SD

*AIC* Akaike information criterion, *BIC* Bayesian information criterion

^a^Male is the reference group

^b^Living with other family structures is the reference group

^c^Trust represents national-level generalized trust

*p < 0.05, **p < 0.01

**Fig. 1:**
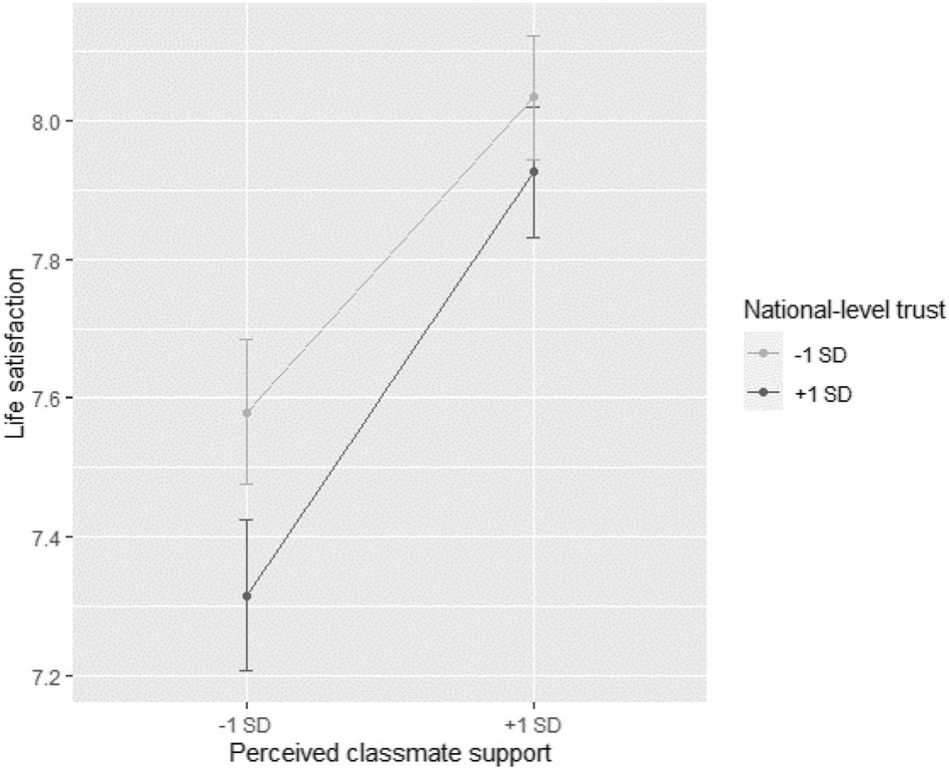
The moderating role of national-level trust in the association between perceived classmate support and adolescent life satisfaction

In Model 2 (Table 3), the best-fitting model with the main and the moderating effects of national-level trust are presented. Life satisfaction was significantly predicted by social support from families (*B* = 0.27*, SE* = 0.02, β = 0.25, *t* = 11.84*, p* < 0.01), friends (*B* = 0.06*, SE* = 0.01, β = 0.05, *t* = 8.45*, p* < 0.01), teachers (*B* = 0.28*, SE* = 0.01, β = 0.14, *t* = 19.82*, p* < 0.01), and classmates (*B* = 0.31*, SE* = 0.02, β = 0.14, *t* = 20.11*, p* < 0.01). Based on the β*-*values, the strength of the association between perceived social support and adolescent life satisfaction was strongest for support from families followed by teachers and classmates, and weakest for support from friends. In addition, the results yield a negative main effect of national-level trust (*B* = *−*0.01*, SE* = 0.00, β = −0.31, *t* = −2.88*, p* < 0.05), indicating that a higher level of national-level trust was associated with lower life satisfaction among adolescents. Also, national-level trust moderated the association between classmate support and adolescent life satisfaction (*B* = 0.00, *SE* = 0.00, *t* = 4.12, *p* < 0.01). No moderating effect emerged for the association between the other three sources of support and adolescent life satisfaction. To further explore the moderation effect, the interaction between classmate support and national-level trust on life satisfaction was plotted. As shown in Fig. 1, the association between perceived classmate support and adolescent life satisfaction was stronger in higher-trust countries than in lower-trust countries.

### Associations Between Social Support and Life Satisfaction by Country/Region

The relative importance of the four sources of support in adolescent life satisfaction was similar across countries/regions (Table 4). Specifically, the association between perceived support from families and adolescent life satisfaction was the strongest in most countries/regions (28 out of 42, 67%), while the association with perceived support from friends was the weakest in most countries/regions (34 out of 42, 81%).

**Table 4:** The Standardized Coefficients (β) of Multivariate Regression Models with Observed Variables across Countries/Regions (N _individual_ = 183,918; N _country_ = 42)

Individual demographic characteristics and all the social support variables were simultaneously added in one model. 95% CI for *B* = 95% confidence interval for unstandardized coefficients. For each country/region, the strongest effect of the source was highlighted in drak grey and the weakest effect of the source was highlighted in light gray. Regarding the relative importance of each sources, the accordingly strongest and the accordingly weakest coefficients were bold

*p < 0.05, **p < 0.01

The associations of each source of support with life satisfaction differed across different countries/regions. Based on βs, the association between adolescent life satisfaction and social support ranged from 0.011 to 0.379 for family support, from −0.070 to 0.142 for friend support, from 0.043 to 0.219 for teacher support, and from 0.058 to 0.232 for classmate support. βs were categorized into three effect sizes, namely negligible in size (β < 0.10), small in size (0.10 < β < 0.30), medium in size (0.30 < β < 0.50), and large in size (β > 0.50) (Cohen, 1992). The association between perceived family support and adolescent life satisfaction was medium in 7 countries/regions, small in 26 countries/regions, and negligible in 9 countries (in which 1 country was non-significant). Regarding perceived friend support, the associations were small in 3 countries and negligible in 39 countries/regions (in which 11 countries/regions were non-significant). In Scotland, perceived friend support was significantly negatively associated with adolescent life satisfaction (β = −0.07*, p* < 0.01). For both perceived teacher and classmate support, the associations in 34 countries/regions were small and 8 were negligible.

## Discussion

Prior research consistently showed that perceived social support is beneficial to life satisfaction, although findings regarding the relative importance of different sources of support for adolescent life satisfaction are inconsistent. Also, research on cross-country differences of the association between support from different sources and life satisfaction in adolescence is scarce and the role of sociocultural factors on the association remains unknown. To enhance the understanding of the importance of perceived social support for life satisfaction in adolescence and to illuminate cross-country variations, the current study investigated the association between perceived social support from different sources and adolescents’ life satisfaction by means of the HBSC study including representative samples of adolescents in 42 countries/regions.

Overall, perceived social support was positively associated with adolescent life satisfaction. In the majority of countries/regions, the association was strongest for support from families followed by teachers and classmates, and weakest for support from friends. Consistent with the expectations, the association between each source of perceived social support and adolescent life satisfaction varied across countries/regions. National-level generalized trust moderated the association between perceived classmate support and adolescent life satisfaction, which was stronger in higher-trust countries/regions than in lower-trust countries/regions. National-level trust did not moderate the associations between the other three sources of support and adolescent life satisfaction.

Replicating existing findings on the importance of perceived social support for adolescent life satisfaction (Proctor et al., 2009), The findings show that support from different sources was, except in one case, positively related to adolescent life satisfaction. They are also consistent with findings showing that parental support is more strongly related to life satisfaction than support from other sources (e.g., Jiménez-Iglesias et al., 2017), and findings showing that families, and parents in particular, remain important providers of support across development (van Harmelen et al., 2016). Extending existing findings, this study shows that the fact that parental support was more important than support from other sources generalized to the majority, but not all, countries/regions. More research is needed to explain why family support may be more important than other sources of support in some countries/regions than others.

The results also highlight the importance of teacher and classmate support. In the majority of countries/regions, teacher and classmate support were important for life satisfaction – albeit somewhat less important than family support – and, in some countries, teacher and classmate support were even more important than family support. From middle childhood into adolescence, peer relationships and the school context gain importance in adolescents’ lives and may serve as a source of support, which contributes to adolescent (social) well-being (Albarello et al., 2018, 2020; Bokhorst et al., 2010; Chu et al., 2010).

Consistent with previous research (e.g., Rueger et al., 2016), the findings show not only that support from classmates was much more important than perceived support from friends, but also that friend support had a negligible association with adolescent life satisfaction and do not show the strongest association with life satisfaction in any of the countries/regions. In Scotland, perceived friend support even shows a significant, but negligible, negative association with adolescent life satisfaction. Possibly, asking early adolescents to rate the support of friends was too general and requires more specific descriptions (e.g., the closest classmate, same-gender friend, or Facebook friend). Research suggested that children define friendship differently (Pössel et al., 2018), and therefore, adolescents in this study may have interpreted what constitutes ’a friend’ in various ways, thereby attenuating the meaning of friend support for life satisfaction.

This study also investigated whether national-level trust moderated the associations between perceived social support from different sources and life satisfaction. The findings show that national-level trust moderated the association between perceived classmate support and adolescent life satisfaction, consistent with the sociocultural model (Vygotsky, 1978). The positive association was stronger in higher-trust countries than in lower-trust countries. Individuals in higher-trust countries are more likely to expect social cooperation, mutual respect, and support from other people, especially from those who have no obligation to help them (Yamagishi, 2011). Consequently, adolescents in higher-trust countries may have higher expectations to receive support from their classmates than adolescents in lower-trust countries. These expectations may make that adolescents in higher-trust countries are more keenly aware of and more sensitive to a lack of classmate support than those in lower-trust countries, which may be negatively related to their life satisfaction.

No other significant cross-level interactions between national-level trust and perceived social support from the other three sources were found (i.e., families, friends, and teachers). Possibly, in early adolescence, national differences in characteristics of proximal social environments (e.g., home, school) might be more important in explaining the association between social support and adolescent life satisfaction than the characteristics of the more distal, national context. For example, parental separation, divorce, and remarriage have been shown to be associated with lower life satisfaction in adolescents (Proctor et al., 2009). Future studies that explore the interplay of factors in the more proximal and national social environment of early adolescents and their respective role in the association between adolescent life satisfaction and perceived social support from different sources would be promising.

The limitations of the present research warrant attention. First, perceived social support from families and friends and perceived social support from teachers and classmates were measured by different scales. To increase internal consistency and comparability across studies, future studies that replicate the findings using the same measures to assess perceived social support from different sources are needed. Second, this study did not differentiate between different types of social support, specifically, emotional, instrumental, informational, and appraisal support (Malecki & Demaray, 2003). Because adolescence is a developmental period characterized by rapid changes in all domains of functioning, and because adolescents become more effective at selecting different sources of social support depending on the type of stressor (Skinner & Zimmer-Gembeck, 2007), different social sources are likely to provide different types of support with different effectiveness over the course of adolescence. Although support from all social sources may be beneficial, research that differentiates between the types of support provided by different sources of support may be promising.

Third, in this study, metric invariance was established which allowed us to compare the association between the four sources of support and life satisfaction across countries/regions. Future studies may establish scalar invariance to reliably compare mean differences across countries (Chen, 2007; Cheung & Rensvold, 2002). Fourth, although individual-level data were obtained separately across three regions (i.e., Scotland, Wales, and England) in the UK and two regions (i.e., Flemish and French language areas) in Belgium, the national data were reported for the UK and Belgium. Given the distinct sociocultural history which has been filled with political strife, there may be regional differences in national-level trust. Relatedly, prior studies suggested that adolescents’ trust in people and institutions is socialized by the quality of their social interactions and experiences (Flanagan & Stout, 2010). Thus, despite of living in the same country, national-level trust may differ across individuals and (sub)groups (e.g., marginalized groups, ethnic minorities). Fifth, cross-sectional data does not allow for causal inferences. Longitudinal studies are needed to further examine the direction of the found associations. Finally, although large and internationally representative samples of early adolescents across 42 countries/regions were used, all participants were from Europe and Canada. In order to paint a more complete picture of the cross-country differences in the association between perceived social support and adolescent life satisfaction, future studies should include more samples from other continents/countries, especially from Africa and Asia.

Despite these limitations, the present study’s findings are useful in advancing the theoretical understanding of the complex association of social support from different sources and adolescent life satisfaction. They may also have practical implications. First and foremost, this research highlights the importance of perceived social support for life satisfaction among early adolescents. The findings highlight the essential role of support in the family and school context for early adolescents’ life satisfaction and underline the importance of policies and intervention strategies to maintain high levels of family support.

The fact that adolescents who perceived low classmate support experienced lower levels of life satisfaction, especially in high-trust countries, underscores the importance of supportive classmates. Safe and supportive classrooms and schools are critical to adolescents’ well-being and educational outcomes. Prior studies have identified effective ways to enhance school safety and personal and social skills of students through various interventions, including schoolwide policies and practices targeting classroom management and positive behavioral interventions (Osher et al., 2010), and social and emotional learning (Durlak et al., 2011). Potentially, such interventions may be more effective in high-trust countries than low-trust countries, but methodologically rigorous studies investigating effectiveness across regions and countries are lacking (Charlton et al., 2020).

## Conclusion

Prior research has established the importance of perceived social support from different sources for life satisfaction in adolescence. Nevertheless, to what extent the association between social support and life satisfaction in early adolescence varied across different social sources and countries remained unclear. Also, the question whether cross-country variations are explained by national-level generalized trust, a sociocultural factor that shapes adolescent socialization remained unanswered. The current study examined the association between perceived social support from different sources and adolescent life satisfaction across 42 countries/regions and explored the moderating role of national-level generalized trust in this association. Consistent with prior studies, perceived social support was mostly positively related to life satisfaction among early adolescents. Nevertheless, the strength of association varied across social sources. For the majority of countries, the association was strongest for support from families, followed by support from teachers and classmates, and weakest for support from friends. The findings on cross-country differences suggest that the effect of sources of support on life satisfaction differed across countries and opens avenues for future cross-cultural research that might further explore factors that may explain cross-cultural differences. In this study, national generalized trust moderated the association between social support and life satisfaction only for classmate support; the positive effect of perceived classmate support on adolescent life satisfaction was stronger in higher-trust countries than in lower-trust countries. Although it is clear that social support is beneficial for life satisfaction in early adolescence, more research is needed to examine the boundary conditions of these results to effectively intervene to promote adolescent well-being across countries.

## Figures and Tables

**Fig. 2:**
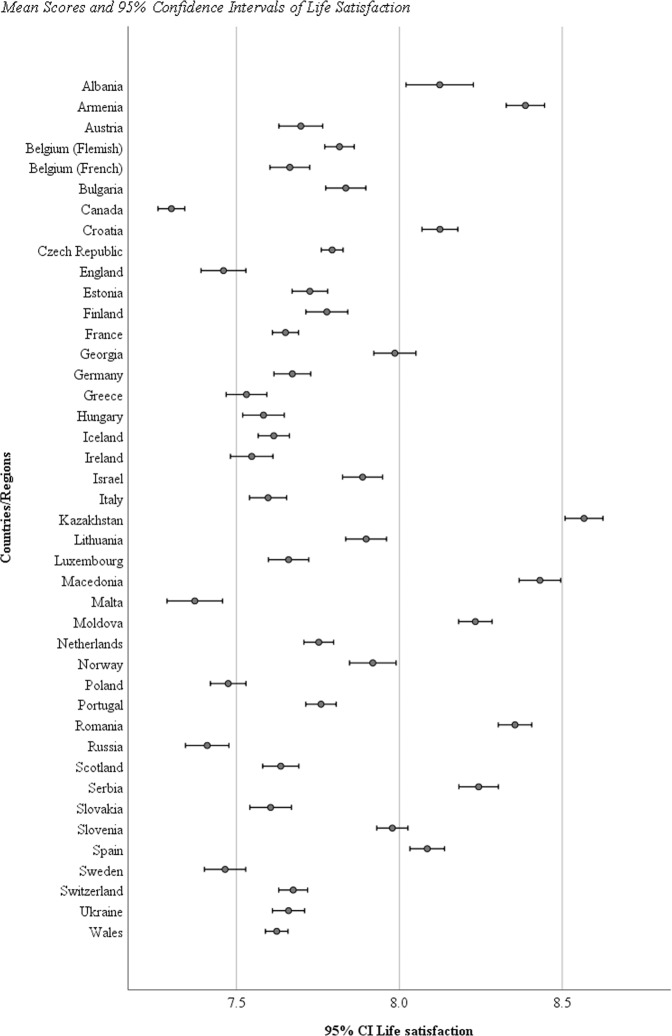
Mean scores and 95% confidence intervals of life satisfaction

**Fig. 3:**
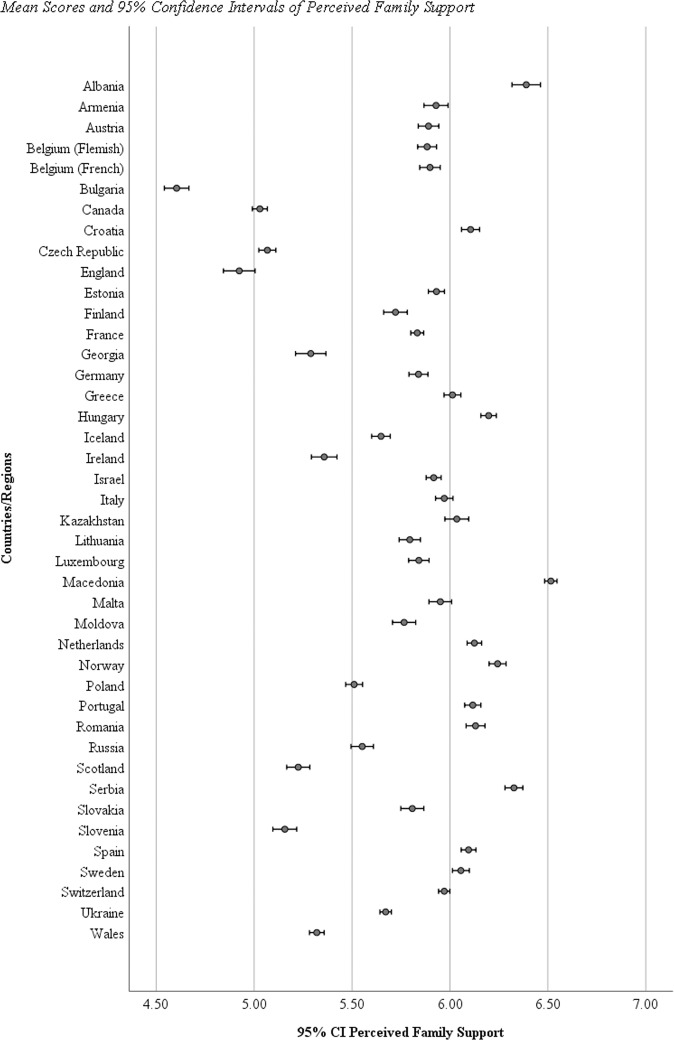
Mean scores and 95% confidence intervals of perceived family support

**Fig. 4:**
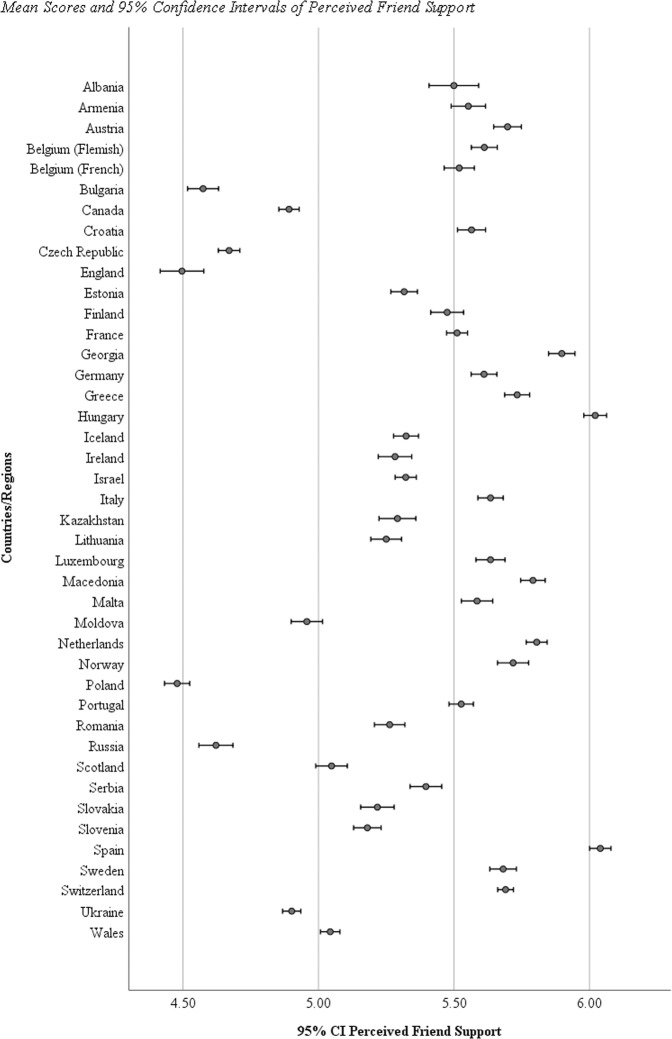
Mean scores and 95% confidence intervals of perceived friend support

**Fig. 5:**
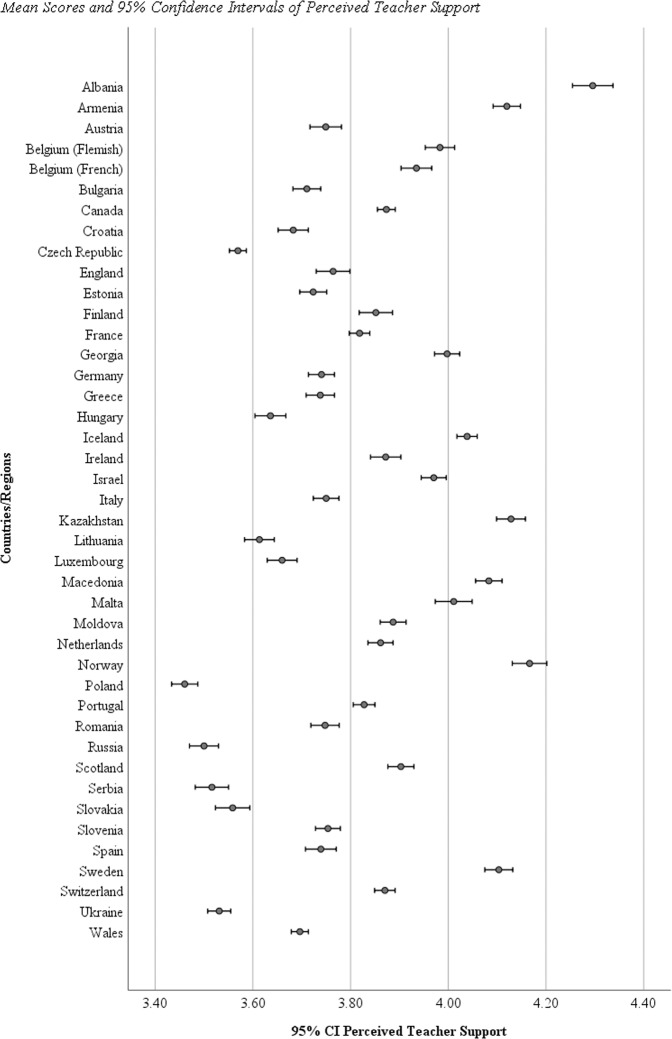
Mean scores and 95% confidence intervals of perceived teacher support

**Fig. 6:**
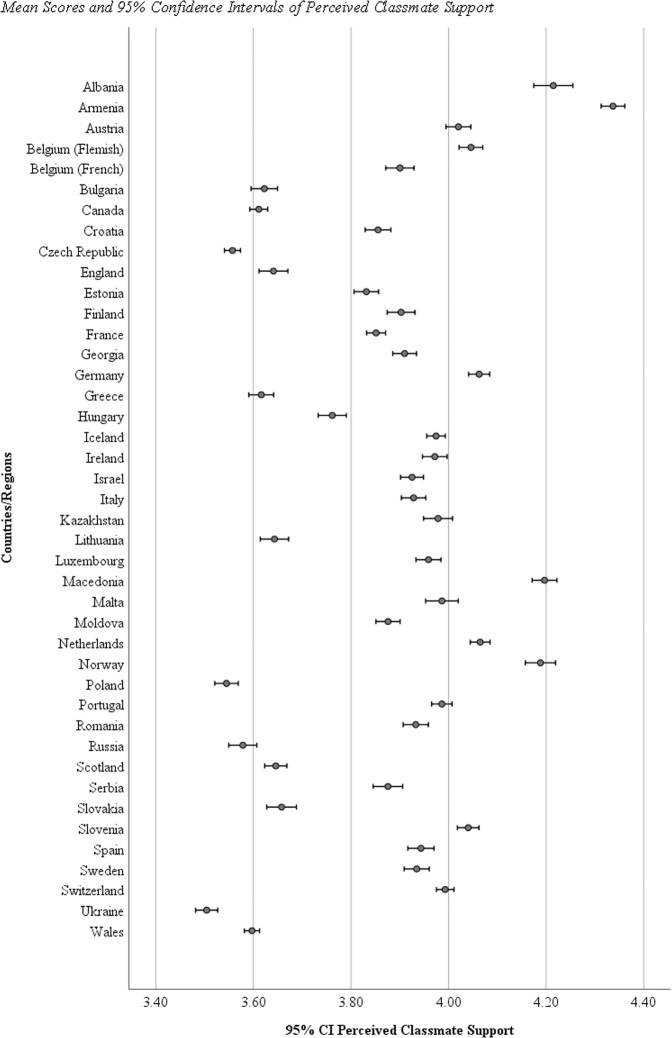
Mean scores and 95% confidence intervals of perceived classmate support

## Appendix 1

Table 5

**Table 5:** Results of testing measurement invariance (N _individual_ = 183,918, N _country_ = 42)

Models	χ^2^	*df*	CFI	TLI	RMSEA		90% CI	*npar*
Baseline Models	44326.797	71	0.973	0.966	0.058	[0.058, 0.059]		48
Configural Model	57132.078	2982	0.968	0.959	0.064	[0.064, 0.065]		2016
Metric Model	69345.852	3392	0.961	0.956	0.067	[0.066, 0.067]		1606
Scalar Model	97042.322	3802	0.945	0.945	0.075	[0.074, 0.075]		1196

*CFI* comparative fit index, *TLI* Tucker–Lewis index, *RMSEA* root mean square error of approximation, *90% CI* the 90% confidence intervals of the RMSEA, *npar* the number of free parameters

## Appendix 2

Results for Model 0 show that the intraclass correlation (ICC) was 0.03, meaning that only 3% of variance in the association between the four sources of perceived social support and life satisfaction was explained at the national-level. In terms of AIC and BIC, the fits for Models 1a–1d (Table 3) were separately compared with the fit of Model 0, and the results show that the values for Model 1a–1d were all smaller than the values of Model 0. Thus, randomizing the slopes of perceived social support from the four sources, that is, family, friends, teachers, and classmates, improved model fit.

Table 6

**Table 6:** Multilevel regression random intercept model (N _individual_ = 183,918, N _country_ = 42)

	Model 0			
	*B*	*SE*	β	*t*-value
Fixed effects (individual-level)				
Gender^a^	−0.21**	0.01	−0.06	−26.98
Age	−0.12**	0.00	−0.11	−49.80
Family structure^b^	0.31**	0.01	0.07	33.93
Family affluence	0.67**	0.01	0.10	48.87
Family support	0.20**	0.00	0.18	80.02
Friend support	0.04**	0.00	0.04	17.68
Teacher support	0.31**	0.00	0.15	63.43
Classmate support	0.35**	0.01	0.15	65.68

	Variance	*S.D*.		
Random parameters				
Between-country variance	0.09	0.30		
Family support				
Friend support				
Teacher support				
Classmate support				
Within-country variance	2.75	1.66		

	Estimate			
Model statistics				
AIC	708259.58			
BIC	708370.93			
Pseudo-R² (total)	0.03			
ICC	0.21			

*AIC* Akaike information criterion, *BIC* Bayesian information criterion

^a^Male is the reference group

^b^Living with other family structures is the reference group

**p* < 0.05, ***p* < 0.01

## Appendix 3

Table 7

**Table 7:** The standardized coefficients of regression models with latent variables across countries/regions

*N*_*individua*l_ = 183,918; *N*_*country*_ = 42. Individual demographic characteristics and all the social support variables were simultaneously added in one model. For each country/region, the strongest effect of the source was highlighted in dark gray and the weakest effect of the source was highlighted in light gray. Regarding the relative importance of each sources, the accordingly strongest and the accordingly weakest coefficients were bold

**p* < 0.05, ***p* < 0.01

## Appendix 4

Figures 2–6
